# Atlas Florae Europaeae notes, 34. Distributions and two conservation profiles of East European species of the *Cytisusratisbonensis* group (Fabaceae)

**DOI:** 10.3897/BDJ.12.e118034

**Published:** 2024-02-23

**Authors:** Alexander Sennikov, Valery N. Tikhomirov

**Affiliations:** 1 University of Helsinki, Helsinki, Finland University of Helsinki Helsinki Finland; 2 Belarusian State University, Minsk, Belarus Belarusian State University Minsk Belarus

## Abstract

**Background:**

The *Cytisusratisbonensis* group (Fabaceae) includes small shrubs with attractive yellow flowers, used in ornamental cultivation. It is widely distributed in southern forest, forest steppe and steppe zones of Eastern Europe, both in the lowlands and low mountains. This group is notorious for its taxonomic complexity and difficulties in identification, which accounted for incongruent taxonomic treatments and common identification errors, and resulted in a poor understanding of the distribution areas. The increasing availability of herbarium collections and accumulation of human observations through digital resources require their critical taxonomic revision and update in order to provide reliable data for plant species mapping, conservation and analysis of distribution areas.

**New information:**

This paper describes a distributional dataset of East European species of the *Cytisusratisbonensis* group, which was prepared for the pan-European grid mapping project, Atlas Florae Europaeae. The taxonomic revision includes seven species and two interspecific hybrids and is based on the critical evaluation of diagnostic characters, nomenclature and synonymy. The territorial scope of this study is Eastern Europe, but it also includes some data from Central Europe, the Caucasus and the neighbouring territories of Siberia and Kazakhstan, which are included in order to trace eastern and south-eastern limits of the species distributions and to establish reliable synonymy. We report 3699 native occurrence records included in the dataset; these records are based on major herbarium collections which are known to hold specimens from Eastern Europe (DNZ, KRA, KRAM, KW, LE, LW, LWKS, MHA, MSK, MSKH, MSKU, MW), complemented by selected specimens from other herbaria (BUNS, CSAU, H, NNSU, NS, NSK, OXF, PVB, RWBG, TUL). The herbarium collections were examined largely *de visu*, but partly online. Besides the herbarium specimens, recognisable photographs documenting human observations on online platforms (florafauna.by, iNaturalist, Plantarium, UkrBIN) were also examined and included. All specimen records are accompanied with textual data and georeferences, which were produced for this dataset. Point and contour distribution maps are created for each accepted species. According to the distributional data, the *Cytisusratisbonensis* group may be classified as temperate lowland and steppic in Eastern Europe. Among European endemic shrubs, *C.cinereus* (as *C.paczoskii*) was assessed in 2017 as Near Threatened, whereas its new IUCN protection status is proposed as Least Concern because the new data demonstrated that its distribution area is much greater than previously believed. A species restricted to the Crimea, *C.wulffii*, is assessed here for the first time as Vulnerable because of its restricted area and small population size (criteria D1,2).

## Introduction

Cytisussect.Tubocytisus DC. (syn. *Chamaecytisus* Link) has been a nightmare of European plant taxonomists. There were various and largely conflicting attempts to delimit this group in Eastern Europe, ranging from rather excessive splitting (Kreczetowicz 1940, Skalická 1983, Tzvelev 1987, Fedoronchuk 2019) to overall lumping (Yakovlev and Svyazeva 1984, Majorov 2014). Broader-scale taxonomic treatments (Cristofolini 1991) presented a rather weighted approach, but suffered from an apparent lack of the representative material and type specimens from Eastern Europe. This controversy stemmed from the difficulties in the interpretation of diagnostic characters in this group, infraspecific variability and recent hybridisation between some species. The small scale of morphological differences suggests a close proximity of many taxa in this section, and the presence of numerous polyploid chromosome counts (e.g. Forissier (1973), Parfionaŭ et al. (1975)) indicates that hybridisation played an important role in the creation of its present taxonomic diversity.

Due to the taxonomic uncertainties, the actual distribution of species in Cytisussect.Tubocytisus is hindered by difficulties in plant identification. This practical trouble leads to the oversimplified representation of the species areas in their distribution maps (e.g. Meusel et al. (1965), Svyazeva and Yakovlev (1986)).

Among the species included in Cytisussect.Tubocytisus, the *C.ratisbonensis* Schaeff. group is the most species-rich and taxonomically complicated in Eastern Europe (Tzvelev 1987). In our work on Fabaceae for Atlas Florae Europaeae, a long-term project of large-scale grid mapping of European vascular plants, we have found that the published records and available herbarium collections of this group are not completely reliable; the group required a new taxonomic revision, and the specimens had to be verified and partly re-identified.

We have produced a new taxonomic scheme for the *Cytisusratisbonensis* group (Sennikov and Tikhomirov 2024a), which is a further development of the system proposed by Cristofolini (1991). For the first time, we have collected ample data that allow us to assess the morphological characters and to make reliable maps for all accepted taxa in the group in Eastern Europe and adjacent territories. With these data at hand, it was possible to produce IUCN global assessments for the taxa occurring in Eastern Europe. So far, only one species of this group, *C.paczoskii* V.I.Krecz. (= *C.cinereus* Host) was assessed as Near Threatened (Sennikov 2017) because of its presumably limited distribution and because of a decline of its habitats. That assessment was based on Didukh (2009) and was considered provisional because of the apparent data deficiency; it is re-assessed here as suggested in the original assessment.

This paper presents a distributional dataset of the *Cytisusratisbonensis* group in Eastern Europe and neighbouring countries, with point maps, which aims to provide the background data for Sennikov and Tikhomirov (2024a). The distributional data are collected for *Atlas Florae Europaeae*, the pan-European grid mapping project (Suominen 1999). This dataset also provides the basis for IUCN assessments of two species involved, which is another objective of the present paper; these make a contribution to the programme of conservation assessments of European endemic shrubs (Wilson et al. 2019).

## Material and methods

### Study area description

The main part of the dataset consists of occurrence records of the *Cytisusratisbonensis* group across Eastern Europe (the former USSR): Belarus, Moldova, Ukraine, Russia and Kazakhstan (European parts up to the watershed of the Ural Mountains). This is the territory covered by Atlas Florae Europaea*e* (Lahti and Lampinen 1999).

The core distribution areas of all species under study are situated within Eastern Europe, but are not limited to this territory, with extensions to Central Europe, the Caucasus and Asia. To circumscribe distribution areas as completely as possible, the East European data were complemented by records from neighbouring territories: largely complete from adjacent territories in the east (Asiatic parts of Russia and Kazakhstan), well-represented from the Caucasus and Transcaucasia (Russian Caucasus, Georgia, adjacent Turkey), and complementary from southern Poland and the Pannonian Plain (Hungary, Romania, Serbia).

With such additions, the presented distributions are deemed complete or nearly complete in the east and south-east, but may be significantly incomplete in the west and south-west where the species records were not available for verification. A special effort was made to trace distributional limits and gaps in Eastern Europe, and we believe that the current information is close to the actual situation in nature. Due to the paucity of available collections from the south-western part of Eastern Europe, this territory is the only part of our study area with the lower level of record density.

### Design description

The dataset was compiled with the aim of making the best representation of native distribution areas, with attention to marginal occurrences which may constitute isolated localities or appear to be new to particular administrative territories or even countries. The second aim was to collect as many records as possible to reveal the core areas of the species distributions and the areas in which the species may be naturally rare.

For historical reasons, herbarium specimens from this extensive territory are organised in major herbarium collections as a single set or as complementing sets, and these collections have been historically studied as a whole and under the same taxonomic paradigm (Tzvelev 1987).

Two major herbaria are included in this study, which are responsible for the whole territory of Eastern Europe: Komarov Botanical Institute, Saint-Petersburg (LE) and Moscow State University (MW; Seregin 2023); however, the density of coverage in these collections is not sufficient for our purposes. These larger subsets of information were complemented by the greatest and most important national or regional collections: Institute of Botany, National Academy of Sciences of Ukraine, Kiev (KW), which covers the whole territory of Ukraine; National University of Lvov (LW) and Institute of Ecology of the Carpathians, National Academy of Sciences of Ukraine, Lvov (LWKS), which cover the territory of western Ukraine; Institute of Experimental Botany, National Academy of Sciences of Belarus, Minsk (MSK), Minsk State University (MSKU) and Central Botanical Garden, Minsk (MSKH), which cover the whole territory of Belorussia; Szafer Institute of Botany, Polish Academy of Sciences, Kraków (KRAM) and Jagiellonian University, Kraków (KRA), which cover south-eastern Poland. Besides, small subsets were added from Main Botanical Garden, Russian Academy of Sciences, Moscow (MHA; Seregin and Stepanova 2020), covering Moscow Region; Tula State Pedagogical University (TUL; Svetasheva and Seregin 2020), covering Tula Region; Central Siberian Botanical Garden, Russian Academy of Sciences, Novosibirsk (NS and NSK; Kovtonyuk et al. 2020), sparsely covering European Russia; Botanical Museum, Finnish Museum of Natural History, University of Helsinki (H), with some specimens from Eastern Europe, Poland, Hungary and the Balkans; Donetsk Botanical Garden (DNZ), covering Donetsk Region; University of Novi Sad, Serbia (BUNS), covering north-western Serbia. Single specimens of distributional or historical importance were added from Crimean Federal University, Simferopol (CSAU), Botanical Garden of the Southern Federal University, Rostov-on-Don (RWBG) and Lobachevsky State University, Nizhny Novgorod (NNSU; Sennikov et al. 2021).

In addition to herbarium specimens, photographs documenting human observations on online platforms were added because of the large extent of such data. We used the following resources: florafauna.by (online data portal of Belorussian wildlife; Florafauna.by 2023), iNaturalist (iNaturalist 2023), with its Russian subset (Seregin et al. 2020), Plantarium (open online atlas and key to plants and lichens of Russia and neighbouring countries; Plantarium 2023), UkrBIN (Ukrainian Biodiversity Information Network; UkrBIN 2023).

### Sampling description

The dataset was prepared during the inventory of herbarium collections and published observations for a new taxonomic revision of the *Cytisusratisbonensis* group (Sennikov and Tikhomirov 2024a).

Herbarium specimens were examined largely *de visu*, by V. Tikhomirov (BUNS, DNZ, KRA, KRAM, KW, LE, LW, LWKS, MHA, MSK, MSKH, MSKU, MW) and A. Sennikov (H). Some minor material was examined on the basis of scanned collections by V. Tikhomirov (NS, NSK, TUL).

The information from herbarium labels was captured and recorded manually, and the georeference data were assigned to each specimen, based on a variety of available current and historical maps. The resulting dataset was prepared on the basis of the Darwin Core template in a spreadsheet of the MS Excel.

Human observations documented by quality photographs were added from online resources. They were harvested from iNaturalist (iNaturalist 2023) by direct export; both Research Grade and lower levels of quality were examined, and the original identifications were corrected when necessary. The data from the other online resources (Florafauna.by 2023, Plantarium 2023, UkrBIN 2023) were entered manually in the same way as for herbarium specimens.

When scanned images of herbarium specimens or observations were available online, links to the original online resources were added to the dataset.

The resulting dataset (4081 occurrences) was made publicly available through Internet Archive (Tikhomirov and Sennikov 2023).

Since the territories outside Eastern Europe were out of the scope of this taxonomic work and sufficient herbarium collections from some territories were largely unavailable, we omitted *C.ratisbonensis* s.str. (which does not occur in Eastern Europe) from consideration. Nevertheless, distribution areas of East European species were traced completely, without territorial exceptions. In Eastern Europe, the least documented territory in international collections was Moldova, for which we partly used published sources when the relevant specimens were unavailable.

### Quality control

Herbarium specimens and documented observations were verified and, if necessary, re-identified according to the taxonomic concept and nomenclature used in Sennikov and Tikhomirov (2024a). Originally, the specimens were identified according to different authorities and taxonomic concepts, in the following herbarium collections: LE (Tzvelev 1987), KW, LW, LWKS (Shevera 1989, Fedoronchuk 2019), MHA, MW, TUL (Majorov 2014), KRA, KRAM (Zieliński 1975), MSK, MSKH, MSKU (Semerenko 1999). This difference led to the practical incompatibility of the material originating from Poland, Ukraine, Belorussia, western and central Russia.

Human observations hosted on iNaturalist were identified according to uncertain sources, but largely using a broad species concept like in Majorov (2014). Among the Russian observations (Seregin et al. 2020), with 1192 records included, we treated 153 records (7.7%) as impossible to identify at the level of species, and 87 records (4.3%) were re-identified. The low number of corrected identifications does not indicate a high quality of the original data because the overwhelming majority of the records belong to *C.ruthenicus*, which is the most common and broadly distributed species in East Europe, and this species name was widely used for the whole group of *C.ratisbonensis* s.l., following Majorov (2014).

Human observations hosted on other online platforms (Florafauna.by 2023, Plantarium 2023, UkrBIN 2023) formed a minor addition to the other material and did not pose any specific problem. This material was critically revised as a whole.

Besides taxonomy, each record was evaluated for the occurrence status. Only native records were taken into account; records of cultivated plants, garden escapes or unintentional dispersal (casual aliens) were disregarded.

### Taxonomic coverage

**Figure 1.**
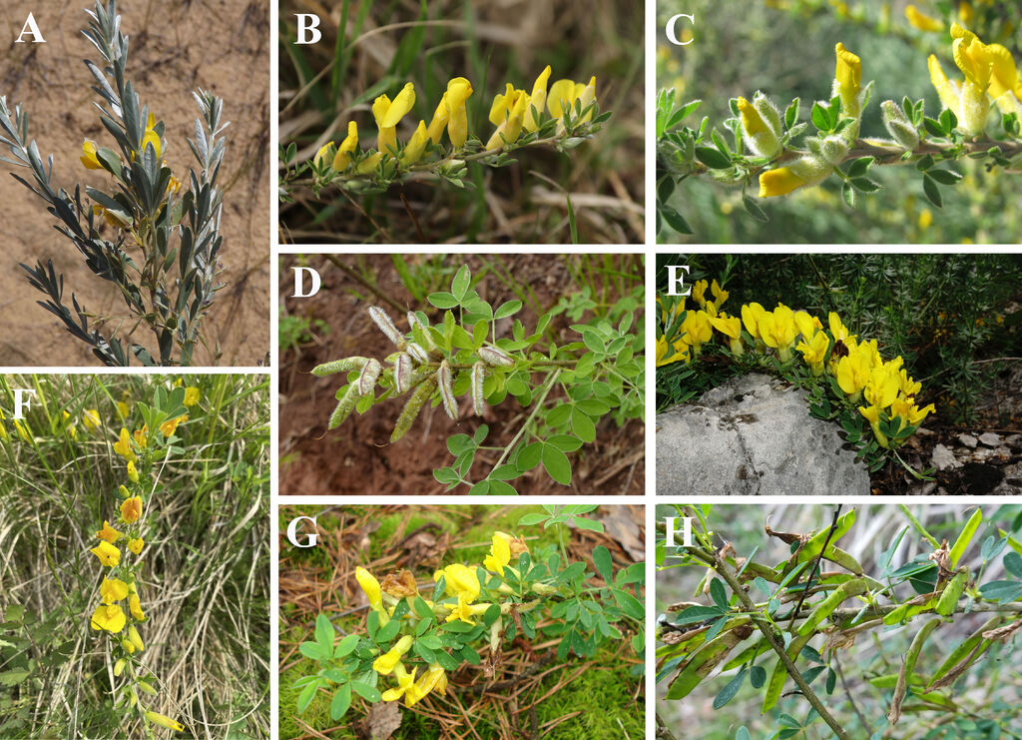
Photo portraits of species in the *Cytisusratisbonensis* group. **A** *C.borysthenicus* Gruner (https://www.inaturalist.org/observations/68954267); **B** *C.ratisbonensis* Schaeff. (https://www.inaturalist.org/observations/191399034); **C** *Cytisuselongatus* Waldst. & Kit. (https://www.inaturalist.org/observations/102192324); **D** *C.cinereus* Host (https://www.inaturalist.org/observations/182044044); **E** *C.wulffii* V.I.Krecz. (https://www.inaturalist.org/observations/153016459); **F** *C.polonicus* Sennikov & Val.N.Tikhom. (https://www.inaturalist.org/observations/115875724); **G** *C.lithuanicus* Gilib. (https://www.inaturalist.org/observations/101929175); **H** *C.ruthenicus* Fisch. ex Otto (https://www.inaturalist.org/observations/168429147). Photo credits: **A** A. Efremov; **B** M. Chytrý; **C** D. Davydov; **D** I. Jovanovic; **E** S. Svirin; **F** T. Suchan; **G** D. Tretjakov; **H** E. Kasandina.

The study is limited to East European members of the *Cytisusratisbonensis* group, which is a group of closely related species of C.sect.Tubocytisus with strictly lateral inflorescences. This group is represented in Eastern Europe by seven species (Fig. 1), namely *C.borysthenicus* Gruner, *C.cinereus* Host (syn. *C.paczoskii* V.I.Krecz.), *C.elongatus* Waldst. & Kit. (syn. *C.lindemannii* V.I.Krecz., *C.triflorus* auct.), *C.lithuanicus* Gilib., *C.polonicus* Sennikov & Val.N.Tikhom., *C.ruthenicus* Fisch. ex Otto and *C.wulffii* V.I.Krecz.; and by two hybrids, i.e. C.×kreczetoviczii Wissjul. and C.×semerenkoanus Sennikov & Val.N.Tikhom. (= *C.czerniaevii* auct.). The taxonomy is obtained from Sennikov and Tikhomirov (2024a) and the nomenclature is updated after Sennikov and Tikhomirov (2024b).

### Species distributions

Species distributions are examined using MaxEnt model software (version 3.4.4) (Phillips et al. 2023) to uncover species-climate interactions for seven species of the *Cytisusratisbonensis* group, which occur in Eastern Europe. Hybrid taxa were not examined because of their co-occurrence with the parental species.

### IUCN assessments

The species assessments follow the Guidelines for Using the IUCN Red List Categories and Criteria (IUCN Standards and Petitions Subcommittee 2022). Area of occupancy and extent of occurrence are measured using the GeoCAT assessment tool (Bachman et al. 2011).

## Species distributions

Although the study area is formally defined as Eastern Europe, the northern limit of the distribution of the *Cytisusratisbonensis* group largely follows the line Minsk – Moscow – Kostroma – Solikamsk.

In the study area, the species of the *Cytisusratisbonensis* group inhabit open places in the southern forest, forest steppe and steppe zones, often occurring on sandy soils and among pine forests. Most of the species are confined to plains or hilly areas and rarely can be found in the lower mountain belt (but *C.ruthenicus* climbs up to 1000 m in the Urals and the Caucasus, and *C.wulffii* is confined to the mountains in the Crimea and occurs at elevations of 600–1200 m).

**Figure 2.**
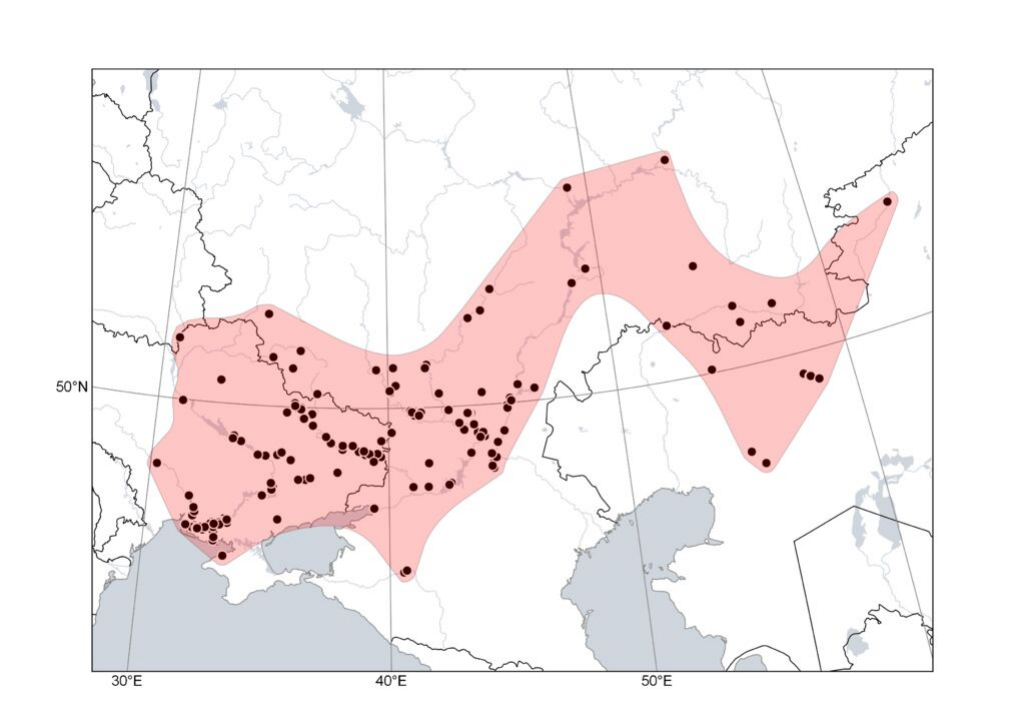
Distribution area of *Cytisusborysthenicus*. Points are based on occurrence records derived from specimens and observations, contours denote the known distribution area.

*Cytisusborysthenicus* (Fig. 2) is widely distributed in the forest steppe and steppe regions in Eastern Europe (Ukraine and Russia) and the neighbouring parts of Asia (Kazakhstan), where it is found at minimal elevations. This strictly psammophilous species occurs in sandy steppes and on denudated sands, on alluvial sands along river sides, sometimes in sparse pine forests on sandy soils.

**Figure 3.**
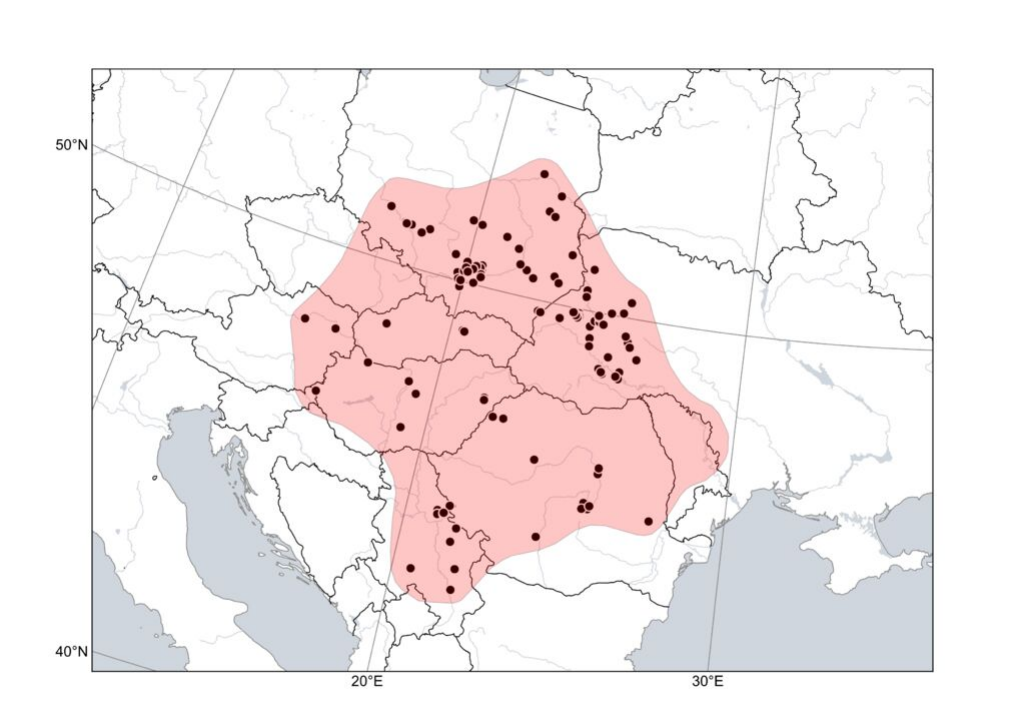
Distribution area of *Cytisuscinereus*. Points are based on occurrence records derived from specimens and observations, contours denote the known distribution area.

*Cytisuscinereus* (Fig. 3) is distributed in hilly lowlands along the north-eastern side of the Carpathians (Poland and Ukraine), together with *C.polonicus*, and in the Pannonian Plain (Serbia, Hungary, Romania; its presence in Slovakia is expected). It is found in open places, meadows and forest margins, often on sandy or calcareous substrates. The species was reported from Moldova and the neighbouring territories of southern Ukraine (Kreczetowicz 1940, Tzvelev 1987, Shabanova et al. 2014); we have not seen any specimens confirming this occurrence.

**Figure 4.**
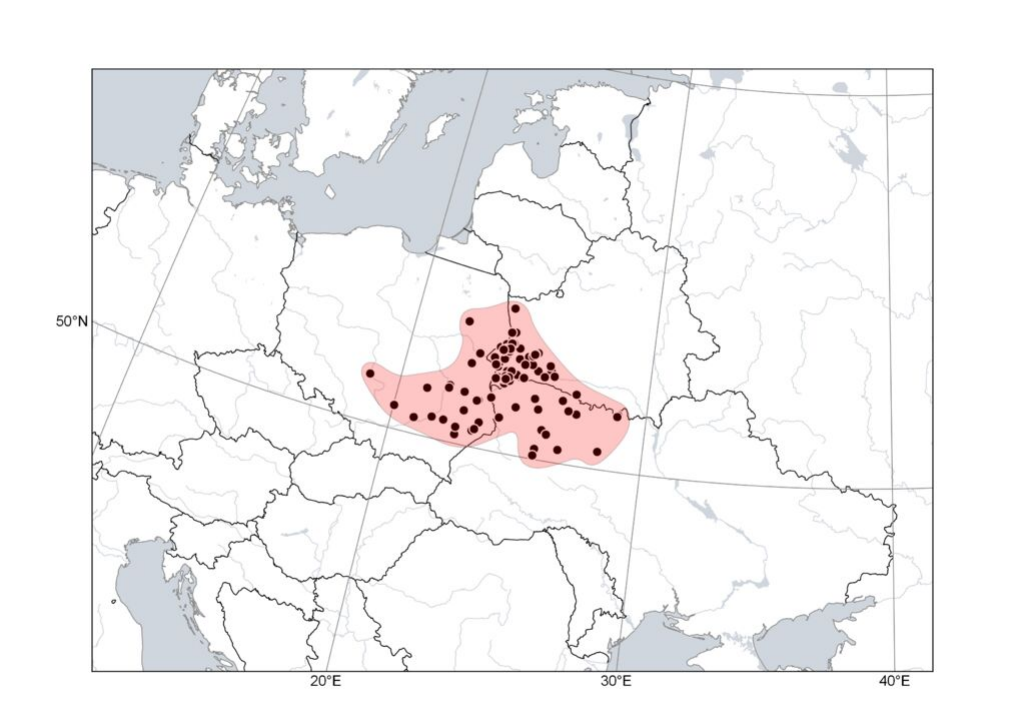
Distribution area of *Cytisuslithuanicus*. Points are based on occurrence records derived from specimens and observations, contours denote the known distribution area.

*Cytisuslithuanicus* (Fig. 4) was recently resurrected for an octoploid segregate of *C.ratisbonensis* s.l. (Sennikov and Tikhomirov 2024a). This species is narrowly distributed mostly in the western Polesie, a flatland belonging to Poland, Belarus and Ukraine, and in neighbouring areas. It occurs in margins of dry pine forests, mostly north-east of the distribution area of *C.polonicus*.

**Figure 5.**
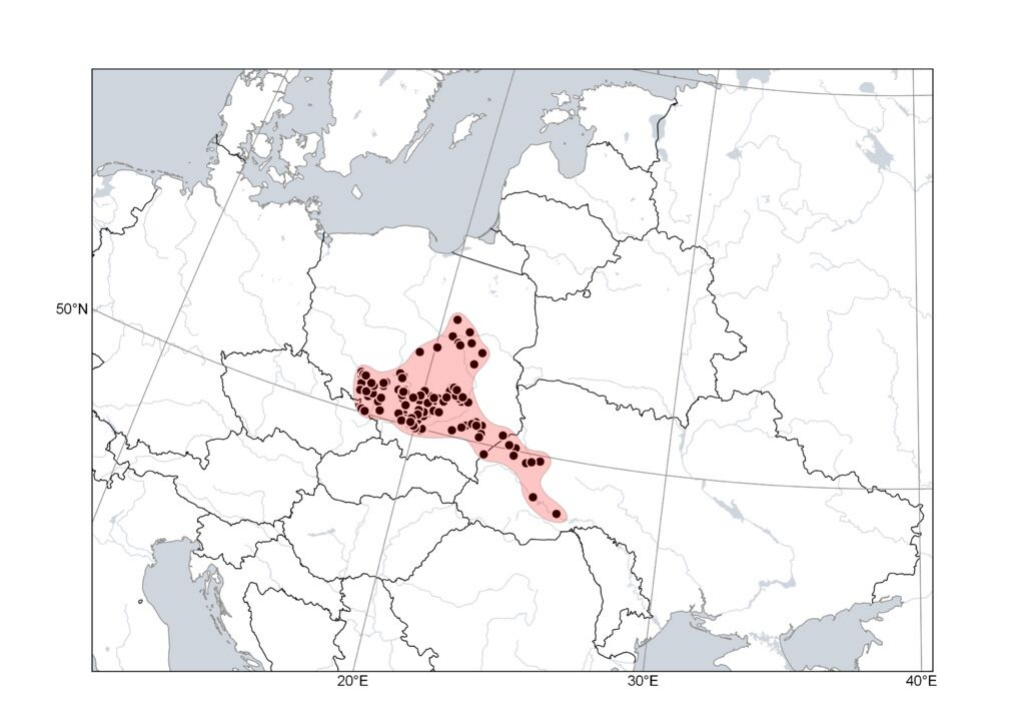
Distribution area of *Cytisuspolonicus*. Points are based on occurrence records derived from specimens and observations, contours denote the known distribution area.

*Cytisuspolonicus* (Fig. 5) was recently segregated from *C.ratisbonensis*, from which it differs in smaller calyces and diploid (vs. tetraploid) chromosome number (Sennikov and Tikhomirov 2024a). This species is rather narrowly distributed in hilly lowlands of Poland and Ukraine, largely along the Carpathians. It occurs at low elevations of 200-400 m on open slopes or calcareous denudations and also in pine forests.

**Figure 6.**
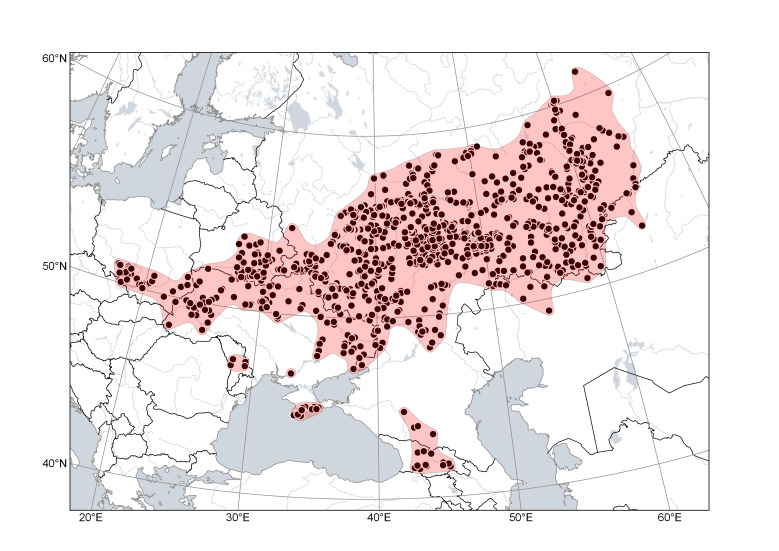
Distribution area of *Cytisusruthenicus*. Points are based on occurrence records derived from specimens and observations, contours denote the known distribution area.

*Cytisusruthenicus* (Fig. 6) is the most widespread and most common species in this group. Its main distribution is centred in the middle and southern parts of the East European Plain, from the Carpathians to the Urals. The species occurs in Poland and Ukraine up to the foothills of the Carpathians; its only verified occurrence in Transcarpathian Ukraine confirms the assumption (Holub and Bertová 1988) that the species should be present also in Slovakia and Hungary. Its occurrence in Moldova assumes its presence in western Romania. In the east, the species distribution includes the southern Urals (with foothills and southern extensions in Russia and Kazakhstan) and the territories along the middle Urals; its easternmost stations are situated in Kurgan Region of Russia and in Qostanay Region of Kazakhstan. In the Crimea, the species occurs mostly along the northern side of the mountains. In the Caucasus, the species is found on the northern side (Russia) and on the southern side, as well as in Transcaucasia (Georgia), where it may occur at elevations of up to 1100 m. The species prefers sparse dry pine forests, shrublands and steppes.

**Figure 7.**
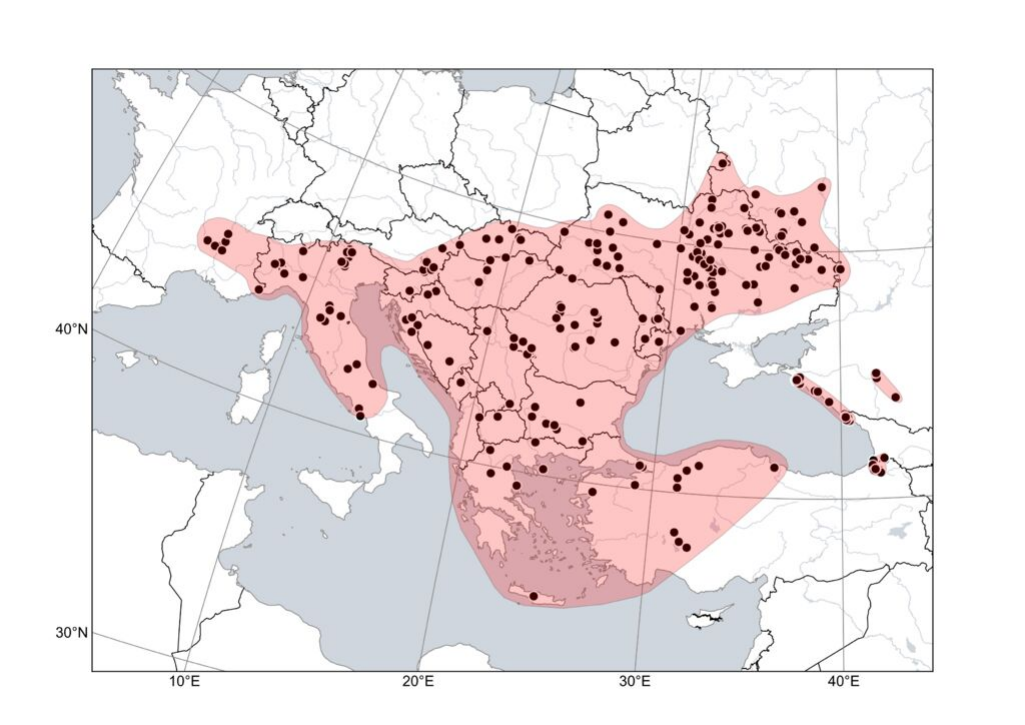
Distribution area of *Cytisuselongatus*. Points are based on occurrence records derived from specimens and observations, contours denote the known distribution area.

*Cytisuselongatus* (Fig. 7) is a thermophilous species which is broadly distributed in Europe and neighbouring Russia. Its core distribution embraces forest steppes of the Podolsk and Dnieper uplands, and other hilly areas and rough terrain over most of Ukraine and neighbouring Moldova and Russia, avoiding the Black Sea lowland. In the west, the species is sparsely distributed in the Carpathians and Balkans, with isolated occurrences in the mountains of northern and central Italy (Cristofolini 1991) and south-eastern France (west of the Rhône River: Tison and de Foucault (2014)). In Asia, the species is nearly confined to the Euxine phytogeographic province as defined by Davis (1985), in Anatolia and the Caucasus, with an extensive presence in the Colchic sector (avoiding the Colchis Lowland) and isolated occurrences in its western part; furthermore, the species was found in the Stavropol Upland. On plains, the species occurs among sparse shrubs in dry creeks, in forest steppes and in oak or pine forests. In mountains, the species is largely confined to lower elevations, but ascends up to 1000-1400 m in Italy and Turkey, with the extreme elevations at 1900 m in the Balkans. In Ukraine, it is frequently found in oak forests and, to a lesser extent, steppe landscapes (Szelag-Sosonko 1987). In the Caucasus, it occurs at elevations below 500(700) m and occupies open slopes and gravelly riverbeds in the areas along the Black Sea; at higher elevations, it is replaced by *C.colchicus* Albov (Portenier and Solod'ko 2002). In Central Europe, the species occurs in margins and openings of xeric thermophilous oak forests, often on calcareous soils.

**Figure 8.**
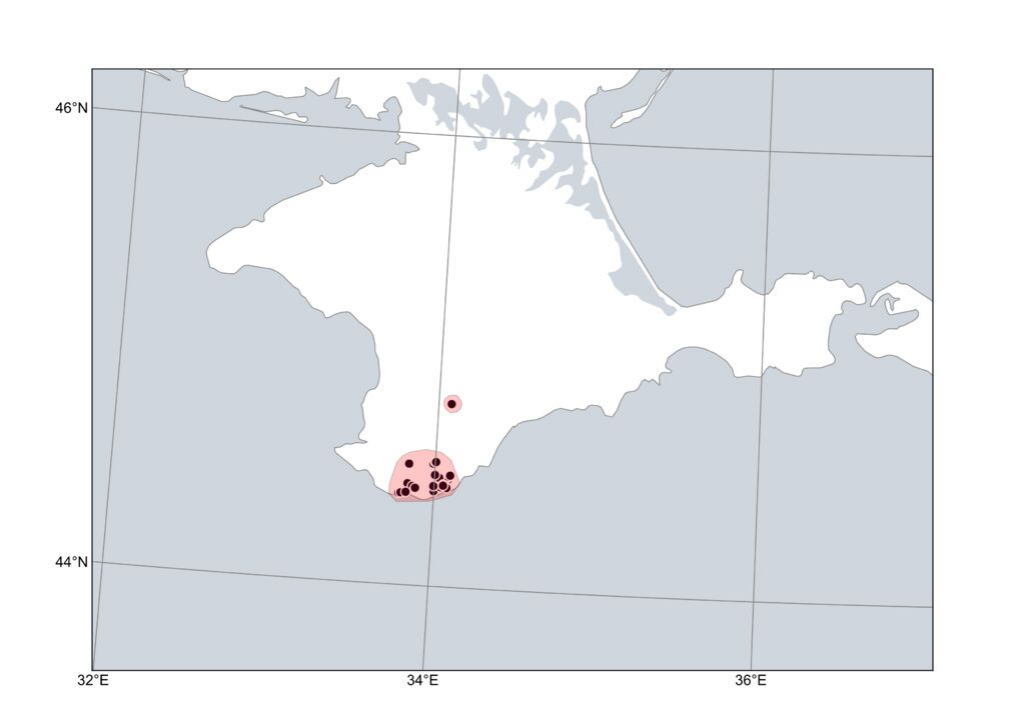
Distribution area of *Cytisuswulffii*. Points are based on occurrence records derived from specimens and observations, contours denote the known distribution area.

*Cytisuswulffii* (Fig. 8) is very narrowly distributed in the mountainous Crimea; its reports from the Western Caucasus (Kreczetowicz 1940, Zernov 2006) are considered erroneous and referable to depauperate individuals of *C.elongatus* (Sennikov and Tikhomirov 2024a). This species occurs on open rocky slopes in pine forests and mountain meadows, often on calcareous substrates.

## Phytogeographical considerations

According to the climate-area interactions, which were uncovered using MaxEnt models created on the basis of complete distribution areas of the species (Table 1), the species of the *C.ratisbonensis* group can be characterised by their areas and climatic preferences in the following way.

*Cytisusborysthenicus* may be considered a true xerophyte species, whose distribution is largely confined to the area of European Pontic steppes and forest steppes (Kajtoch et al. 2016). Two species (*C.cinereus*, *C.polonicus*) are linked to the area of xeric grasslands of south-eastern Poland and north-western Ukraine (Kajtoch et al. 2016), largely extending to forest steppes of the Pannonian Basin (*C.cinereus*). *Cytisuselongatus* can be considered thermophilous and linked to southern types of oak forests. These four species are controlled by temperature parameters, but also are largely dependent on the precipitation regime.

The distributions of two species, *C.lithuanicus* and *C.ruthenicus*, are controlled by temperature parameters, but to a lesser extent by precipitation. Their distributions are linked to southern types of pine forests and only to a minor extent overlap with the area of *C.elongatus*.

These six species have different relationships to the temperature evenness and climatic seasonality. *Cytisuscinereus*, *C.lithuanicus* and *C.polonicus* occur along or within the chain of the Carpathians; these species require a climate featuring lesser seasonal fluctuations in temperature and moisture. On the other hand, *C.borysthenicus*, *C.elongatus* and *C.ruthenicus* have more eastern and southern occurrences; they are less dependent on the climatic evenness and may survive during hot and dry seasons in more arid or continental areas.

Among the floristic elements delimited by Finnie et al. (2007), *Cytisusborysthenicus* falls within the *Dianthuscapitatus*-element, which is largely congruent with steppic plants. The other species seemingly fall within the *Lychnisflos-cuculi* element, which is defined broadly to include many plants widely or more narrowly distributed in Central Europe, Balkans and the southern part of Eastern Europe.

Although the species of the *C.ratisbonensis* group are characterised by rather subtle differences in pubescence, their distributions follow certain phytogeographic and climatic patterns, thus indicating the utility of narrowly defined taxa in plant geography.

## Species Conservation Profiles

### Cytisus cinereus

#### Species information

Scientific name: Cytisuscinereus

Species authority: Host

Synonyms: *Cytisushorniflorus* Borbás, *Cytisuspaczoskii* V.I.Krecz., *Chamaecytisuspaczoskii* (V.I.Krecz.) Klásk.

Common names: Ракитник серый (Russian), Ракитник Пачоского (Russian), Зіновать Пачоського (Ukrainian)

Kingdom: Plantae

Phylum: Tracheophyta

Class: Magnoliopsida

Order: Fabales

Family: Fabaceae

Taxonomic notes:

This species belongs to the *Cytisusratisbonensis* group (Cytisussect.Tubocytisus DC.), which includes up to 10 closely related taxa with the main distribution area in Eastern Europe. This group has been poorly studied and remains unsatisfactorily resolved in Central Europe and especially in the Balkans.

The taxonomy of this species was poorly understood. It was considered endemic to Eastern Europe until Sennikov and Tikhomirov (2024a) found that this species is widely distributed also in south-eastern Poland and in the whole of the Pannonian Plain, from which it was described at least twice. The earliest of these two names, *C.cinereus* Host, provides the correct name for the species.

Region for assessment: EuropeGlobal

#### Editor & Reviewers

##### Reviewers

Reviewers: Allen, D.J.

##### Editor

Editor: Sennikov, A.N. & Tikhomirov, V.N.

#### Geographic range

Biogeographic realm: Palearctic

Countries: PolandRomaniaAustriaHungaryMoldovaSerbiaUkraine

Map of records (Google Earth): Suppl. material 1

Basis of EOO and AOO: Observed

Basis (narrative): The species distribution has been incompletely studied (Sennikov 2017), but can be considered sufficiently known now. Based on the data specifically collected for Atlas Florae Europaeae, the distribution area of the species, as assessed via GeoCAT, has an estimated extent of occurrence (EOO) of ca. 625,000 km^2^ and an area of occupancy (AOO) of ca. 485 km^2^. The area of occupancy is probably still underestimated because the current coverage of species records may be considerably incomplete in the southern part of its distribution area.

Min Elevation/Depth (m): 100

Max Elevation/Depth (m): 500

Range description:

The existing data indicate that the species is rather broadly distributed in Central Europe, including hilly lowlands north and north-east of the Carpathians and the Pannonian Plain with adjacent foothills. Its previous treatment as endemic to Ukraine and Moldova (Tzvelev 1987, Didukh 2009) is no longer supported.

The occurrence in Moldova (Tzvelev 1987) has not been verified because of the lack of relevant herbarium specimens. It can be provisionally accepted because of one confirmed locality in Romania that is situated within the same area of true steppes as in Moldova (Kajtoch et al. 2016).

#### Extent of occurrence

EOO (km2): 625,000

Trend: Stable

Justification for trend:

There is no change in the total distribution area known for this species, hence the trend is considered stable. Although many species localities have been under high anthropogenic pressure, we are not aware of complete extinctions in any large part of the species distribution.

The earlier estimation of the species extent of occurrence (ca. 77,440 km^2^) (Sennikov 2017) was eight times lower because the species was considered endemic to Ukraine and Moldova and the western part of its distribution area remained unknown.

Causes ceased?: No

Causes understood?: Yes

Causes reversible?: No

Extreme fluctuations?: No

#### Area of occupancy

Trend: Decline (observed)

Justification for trend: Species populations are under constant anthropogenic pressure because of habitat destruction: clear-cutting of sparse forests, ploughing of steppic lands, mining of limestone. The greatest negative impact is estimated to have happened in the past, when forests were less protected and steppes were actively converted into arable lands; this change is considered practically irreversible. The current trend remains slightly negative because of the continuous, albeit less destructive, anthropogenic impact.

Past decline (%): 10

Future decline (%): 5

Causes ceased?: No

Causes understood?: Yes

Causes reversible?: No

Extreme fluctuations?: No

AOO (km2): 485

#### Locations

Number of locations: 6

Justification for number of locations: The species has a very broad distribution, occurring in six countries. Since the major threat to the species is considered to come from the loss and degradation of habitats, which result from the anthropogenic pressure, we define the number of locations as the number of jurisdictions in which similar regulations apply (IUCN Standards and Petitions Subcommittee 2022). However, the occurrence in individual countries (like Poland or Ukraine) is so extensive that a single event driving it to extinction seems to be practically impossible.

Trend: Decline (observed)

Justification for trend: There is a loss of habitats recorded for this species in many countries because much damage commonly occurred to steppes and xeric areas in the past (when steppes were converted to arable lands and forests were logged) and still occurs now (because of invasions of alien species, habitat fragmentation, recreation and other human impact).

Extreme fluctuations?: No

Justification for extreme fluctuations: No fluctuations are known in this species group.

#### Population

Number of individuals: Many, presumably well over 10000.

Trend: Decline (inferred)

Justification for trend: The species is likely declining in some parts of its distribution area because of the ongoing loss and degradation of habitats. The global population should still be abundant, with many thousands of mature individuals.

Basis for decline: (c) a decline in area of occupancy, extent of occurrence and/or quality of habitat

Causes ceased?: No

Causes understood?: Yes

Causes reversible?: No

Extreme fluctuations?: No

Justification for extreme fluctuations: No extreme fluctuations are known for this species group due to its long generation length.

#### Subpopulations

Trend: Decline (inferred)

Justification for trend: Ongoing loss and degradation of habitats may destroy certain subpopulations.

Extreme fluctuations?: No

Justification for extreme fluctuations: No fluctuations are known in this species group.

Severe fragmentation?: No

Justification for fragmentation: The main distribution area is essentially continuous in lowlands and seems to consist of three large subareas (subpopulations), in the north and south of East Europe and in the Pannonian Plain. Within the subareas, genetic exchange may be significantly hampered by habitat fragmentation. The subpopulations are significantly large and not at the risk of rapid extinction.

#### Habitat

System: Terrestrial

Habitat specialist: Yes

Habitat (narrative): The species occurs in lowlands (plains and hilly uplands) in open places and meadow steppes, sparse shrublands, sparse dry forests, often on sandy or calcareous soils. It is strictly confined to lower elevations, below 500 m. In Central Europe, the species occurs in the Pannonian Plain, in margins and openings of xeric thermophilous oak forests, steppe-like meadows and sandy areas, slightly ascending to the mountains by larger river valleys.

Trend in extent, area or quality?: Decline (observed)

Justification for trend: We consider the species habitat extent and quality declining due to the common degradation of suitable habitats (reduction and deterioration of oak forests, steppes and steppe-like landscapes) in Europe.

##### Habitat

Habitat importance: Major Importance

Habitats: 1.4. Forest - Temperate3.4. Shrubland - Temperate4.4. Grassland - Temperate

#### Ecology

Size: Up to 60(80) cm

Generation length (yr): 10

Dependency of single sp?: No

Ecology and traits (narrative): This species is a small multi-stemmed shrub 30-60(80) cm tall, with long branches and abundant flowers; generation length 10-15(20) years. The leaves are glabrous above and appressed-pilose below. The flowers are intensely yellow, possibly with a purple tint in the middle, with a glabrous standard. Seed set is abundant, vegetative reproduction absent. The species typically occurs in small groups, not forming extensive stands.

#### Threats

Justification for threats: Major threats to the species are loss and fragmentation of habitats (most commonly conversion of meadows, forest steppes and shrublands into agricultural lands), destruction of habitats (road construction, quarries in limestone areas) and invasions of alien plant species. In spite of the apparent reduction of habitats and, likely, the number of individuals in various localities, species survival is still not in danger because of its large distribution and numerous populations.

##### Threats

Threat type: Ongoing

Threats: 1. Residential & commercial development2.1. Agriculture & aquaculture - Annual & perennial non-timber crops4. Transportation & service corridors8. Invasive and other problematic species, genes & diseases

#### Conservation

Justification for conservation actions:

The species is protected in some countries with its habitats, i.e. steppes and thermopholous oak forests. In Ukraine, it occurs in the Medobory Strict Nature Reserve and in a number of smaller protected territories, and is legally protected (included in the national Red Data Book) as Rare because of its formerly (sub)endemic status and limited occurrence (Didukh 2009).

The species has been assessed as Near Threatened (NT) in the IUCN Red List (Sennikov 2017). This assessment stressed that the species should be re-assessed when further information becomes available.

Based on the new data that revealed its much larger extent of occurrence and many further localities recorded in several countries, we suggest to re-assess the global and European conservation status of the species as Least Concern (LC). The species may be protected at the national level because of its limited occurrence and a high sensitivity of its habitats (steppe-like and sandy open habitats and sparse thermophilous forests) to human impact, as implemented in Ukraine (Didukh 2009) and suggested in Moldova (Shabanova et al. 2014). Habitat losses due to agriculture and developoment are a major threat to European plants (Hochkirch et al. 2023); since this factor also poses a major threat to *C.cinereus*, its populations require monitoring and protection as part of the endangered steppic landscape and its biodiversity (Kajtoch et al. 2016).

##### Conservation actions

Conservation action type: In Place

Conservation actions: 1.1. Land/water protection - Site/area protection

### Cytisus wulffii

#### Species information

Scientific name: Cytisuswulffii

Species authority: V.I.Krecz.

Synonyms: *Chamaecytisuswulffii* (V.I.Krecz.) Klásk.

Common names: Ракитник Вульфа (Russian), Зіновать Вульфа (Ukrainian)

Kingdom: Plantae

Phylum: Tracheophyta

Class: Magnoliopsida

Order: Fabales

Family: Fabaceae

Taxonomic notes:

This species belongs to the *Cytisusratisbonensis* group (C.sect.Tubocytisus DC.) and differs from the other species by its life form (small prostrate shrubs with abundantly branching stems) and appressed pubescence on its calyces and both sides of its leaflets.

This species has been rather recently separated from *C.polytrichus* M.Bieb., which differs in its patent pubescence and belongs to the *C.hirsutus* group (Cristofolini 1991); both species co-occur in the same territory. *Cytisuswulffii* was considered present in the north-western Russian Caucasus (e.g. Zernov (2006)), but Tzvelev (1987) treated it as endemic to the Crimea. Sennikov and Tikhomirov (2024a) referred the Caucasian records to *C.elongatus*, which differs by erect to suberect stems and subpatent pubescence of its calyces.

The species epithet was sometimes misspelled as "wulfii"; its correct spelling "wulfii" is determined by the protologue (Kreczetowicz 1940).

Region for assessment: EuropeGlobal

#### Editor & Reviewers

##### Reviewers

Reviewers: Allen, D.J.

##### Editor

Editor: Sennikov, A.N. & Tikhomirov, V.N.

#### Geographic range

Biogeographic realm: Palearctic

Countries: Ukraine

Map of records (Google Earth): Suppl. material 2

Basis of EOO and AOO: Observed

Basis (narrative):

As assessed via GeoCAT, the species has an estimated extent of occurrence (EOO) of ca. 600 km^2^ and an area of occupancy (AOO) of ca. 30 km^2^. Its distribution is largely compact and continuous and supported by many records and observations. As a result of so many records, both in recent and historical times, we assume that the known distribution area is practically complete.

The AOO is estimated on the basis of the cell width of 1 km^2^, which can still be an overestimation taking into account the small population size and the limited distribution of suitable habitats.

Min Elevation/Depth (m): 600

Max Elevation/Depth (m): 1250

Range description: The species distribution is confined to the Crimea; its records from the Caucasus (e.g. Zernov (2006)) are not accepted. Based on our data obtained from herbarium collections, the species occurs in the mountains mostly between Foros and Yalta, with a few occurrences further north (Tikhomirov and Sennikov 2023). The northern occurrences were previously neglected (Didukh 2009, Yena and Fateryga 2015). The northernmost, rather isolated record from Partizanskoe (formerly Sabla) is based on a single specimen, which is 200 years old, and should be confirmed.

#### Extent of occurrence

EOO (km2): 600

Trend: Stable

Justification for trend: The species has been observed many times since its first collections in the beginning of the 19^th^ century. Many localities have been confirmed lately, and there are no reports of any significant decline. However, the species is under continuous anthropogenic pressure due to extensive recreational activities.

Causes ceased?: No

Causes understood?: Yes

Causes reversible?: Yes

Extreme fluctuations?: No

Justification for extreme fluctuations: No fluctuations have been reported for this species.

#### Area of occupancy

Trend: Stable

Justification for trend: No apparent decline has been observed or inferred for the species in spite of some anthropogenic pressure caused by recreation.

Causes ceased?: No

Causes understood?: Yes

Causes reversible?: Yes

Extreme fluctuations?: No

Justification for extreme fluctuations: No fluctuations are known.

AOO (km2): 30

#### Locations

Number of locations: 1

Justification for number of locations: The species has a small and continuous distribution area, which is homogeneous in landscape and human management. We assume that the ongoing threats (anthropogenic pressure, mostly recreation) are the same for the whole distribution area and evenly affect its whole territory.

Trend: Stable

Justification for trend: No decline has been observed or reported.

Extreme fluctuations?: No

Justification for extreme fluctuations: No fluctuations are known.

#### Population

Number of individuals: 500

Trend: Unknown

Justification for trend: The population size and dynamics have not been explored yet. On the basis of the known number of localities and the small size of local populations, we assume that the species may be represented by ca. 500 mature individuals. The trend is completely unknown, but may be rather stable, since no apparent degradation or disappearance of local populations has been reported. However, due to continuous anthropogenic pressure caused by recreation, some decline may potentially happen in the future.

Causes ceased?: No

Causes understood?: Yes

Causes reversible?: Yes

Extreme fluctuations?: No

Justification for extreme fluctuations: No fluctuations are known.

#### Subpopulations

Trend: Stable

Justification for trend: No decline in the species occurrence has been recorded.

Extreme fluctuations?: No

Justification for extreme fluctuations: No fluctuations have been observed.

Severe fragmentation?: No

Justification for fragmentation: The distribution area is largely continuous.

#### Habitat

System: Terrestrial

Habitat specialist: Yes

Habitat (narrative): The species is confined to the middle and upper belts of the Crimean mountains, where it occurs in meadows and open places among pine forests. It prefers open rocks, often calcareous outcrops, and seems to be a petrophyte.

Trend in extent, area or quality?: Stable

Justification for trend: So far, there is no information regarding any extensive destruction of the species habitats. In spite of intensive recreational pressure, which occurs in some places, the species distribution area is largely covered by legally protected territories.

##### Habitat

Habitat importance: Major Importance

Habitats: 6. Rocky areas (e.g. inland cliffs, mountain peaks)

##### Habitat

Habitat importance: Suitable

Habitats: 4.4. Grassland - Temperate

#### Ecology

Size: 5-20 cm

Generation length (yr): 10

Dependency of single sp?: No

Ecology and traits (narrative): This species is a very small prostrate shrub 5-20 cm above ground, with many thin branches; generation length estimated at 10-15 years. The leaves are appressed-pilose on both sides. The flowers are intensely yellow, with a glabrous standard. Flowering is abundant, seed set unknown, vegetative reproduction absent. The species typically occurs in small groups, rather than forming extensive mats.

#### Threats

Justification for threats: At present, the main threat for the species is recreation. Human impact on the Crimean mountains is rather high, with many visitors coming annually. Trampling and further deterioration of habitats may be the main factor.

##### Threats

Threat type: Ongoing

Threats: 6.1. Human intrusions & disturbance - Recreational activities

#### Conservation

Justification for conservation actions:

The species is legally protected in the Crimea. It is included in the Red Data Book of Ukraine as Vulnerable (Didukh 2009) and in the Red Data Book of the Crimea as Rare (Yena and Fateryga 2015) because of its (sub)endemic status and limited occurrence. It occurs in the Crimean Strict Nature Reserve and in the Yalta Mountain Forest Strict Nature Reserve.

Based on the limited area of occurrence (presumably less than 30 km^2^), low population size (less than 1000, but likely 500 mature individuals) and number of locations (single), we suggest to assess the global and European conservation status of the species as Vulnerable (VU; criteria D1,2).

##### Conservation actions

Conservation action type: In Place

Conservation actions: 1.1. Land/water protection - Site/area protection

## Supplementary Material

10316871-911C-5B1A-AF5D-C9C41FB5A733 10.3897/BDJ.12.e118034.suppl1 Supplementary material 1 Distribution of *Cytisuscinereus* as viewed via GeoCAT Data type occurrences File: oo_953518.kml https://binary.pensoft.net/file/953518 Tikhomirov, V.N. & Sennikov, A.N.

3E0F9CE9-FE85-57CE-AF81-A9986E408BAC 10.3897/BDJ.12.e118034.suppl2 Supplementary material 2 Distribution of Cytisuswulffii as viewed via GeoCAT Data type occurrences File: oo_515752.kml https://binary.pensoft.net/file/515752 Tikhomirov, V.N. & Sennikov, A.N.

## Figures and Tables

**Table 1.** Bioclimatic variables used for the MaxEnt model and their contributions (in percent). Figures in bold denote the leading contributions.

Code	Variable Description	*Cytisusborysthenicus*	*Cytisuscinereus*	*Cytisuselongatus*	*Cytisuslithuanicus*	*Cytisuspolonicus*	*Cytisusruthenicus*	*Cytisuswulffii*
BIO1	Annual Mean temperature	0.1	6.1	10.3	1.9	**16.7**	1.7	0
BIO2	Mean Diurnal Range	0.3	3.1	0.1	1.5	2.8	0.4	0.9
BIO3	Isothermality	1.1	**21.9**	9.1	**31.7**	5.4	0.2	9.3
BIO4	Temperature Seasonality	**15.3**	5	11.3	0	0.1	10.5	0.6
BIO5	Max Temperature of Warmest Month	0.2	0	3.6	0	0.4	**16.7**	0
BIO6	Min Temperature of Coldest Month	0.9	0	0	0.1	0.1	5.6	0
BIO7	Temperature Annual Range	0.4	2.1	6.6	0.1	8.6	0.7	0.3
BIO8	Mean Temperature of Wettest Quarter	**31.4**	10.1	1	**15.5**	6.7	6.5	**49.2**
BIO9	Mean Temperature of Driest Quarter	0.2	0.3	0.7	0.1	1.8	10.8	0
BIO10	Mean Temperature of Warmest Quarter	**16.3**	0.4	**28.2**	0	0	**19.7**	0
BIO11	Mean Temperature of Coldest Quarter	0.2	0.3	0	7.7	0	0	**22.3**
BIO12	Mean annual precipitation	0	0.3	0.1	0	0	0.4	6.8
BIO13	Precipitation of Wettest Month	0	0.7	0	0	0.1	0.2	0
BIO14	Precipitation of Driest Month	10.2	0.9	0.8	0.3	0.3	0.1	2
BIO15	Precipitation Seasonality	10.3	**19.1**	1.1	9.6	**32.1**	0.3	5.5
BIO16	Precipitation of Wettest Season	0.1	0	0.1	0	0	0	0
BIO17	Precipitation of Driest Season	0.4	0	3.8	0.2	0.1	0	0
BIO18	Precipitation of Warmest Quarter	0.2	**28.6**	**22.2**	**19.2**	**24.1**	**25.9**	2.9
BIO19	Precipitation of Coldest Quarter	1.4	0	0.4	0	0.5	0.1	0
	Altitude	11	0.9	0.5	12.1	0.2	0.1	0
